# Network Service and Resource Orchestration: A Feature and Performance Analysis within the MEC-Enhanced Vehicular Network Context

**DOI:** 10.3390/s20143852

**Published:** 2020-07-10

**Authors:** Nina Slamnik-Kriještorac, Erik de Britto e Silva, Esteban Municio, Henrique C. Carvalho de Resende, Seilendria A. Hadiwardoyo, Johann M. Marquez-Barja

**Affiliations:** IDLab—Faculty of Applied Engineering, University of Antwerp—imec, Sint-Pietersvliet, 2000 Antwerp, Belgium; Erik.deBrittoeSilva@uantwerpen.be (E.d.B.e.S.); esteban.municio@uantwerpen.be (E.M.); Henrique.CarvalhoDeResende@uantwerpen.be (H.C.C.d.R.); seilendria.hadiwardoyo@uantwerpen.be (S.A.H.); johann.marquez-barja@uantwerpen.be (J.M.M.-B.)

**Keywords:** 5G, control, edge and cloud computing, MANO, MEC, monitoring, NFV, orchestration, V2X, vehicular communications

## Abstract

By providing storage and computational resources at the network edge, which enables hosting applications closer to the mobile users, Multi-Access Edge Computing (MEC) uses the mobile backhaul, and the network core more efficiently, thereby reducing the overall latency. Fostering the synergy between 5G and MEC brings ultra-reliable low-latency in data transmission, and paves the way towards numerous latency-sensitive automotive use cases, with the ultimate goal of enabling autonomous driving. Despite the benefits of significant latency reduction, bringing MEC platforms into 5G-based vehicular networks imposes severe challenges towards poorly scalable network management, as MEC platforms usually represent a highly heterogeneous environment. Therefore, there is a strong need to perform network management and orchestration in an automated way, which, being supported by Software Defined Networking (SDN) and Network Function Virtualization (NFV), will further decrease the latency. With recent advances in SDN, along with NFV, which aim to facilitate management automation for tackling delay issues in vehicular communications, we study the closed-loop life-cycle management of network services, and map such cycle to the Management and Orchestration (MANO) systems, such as ETSI NFV MANO. In this paper, we provide a comprehensive overview of existing MANO solutions, studying their most important features to enable network service and resource orchestration in MEC-enhanced vehicular networks. Finally, using a real testbed setup, we conduct and present an extensive performance analysis of Open Baton and Open Source MANO that are, due to their lightweight resource footprint, and compliance to ETSI standards, suitable solutions for resource and service management and orchestration within the network edge.

## 1. Introduction

**Figure 1:**
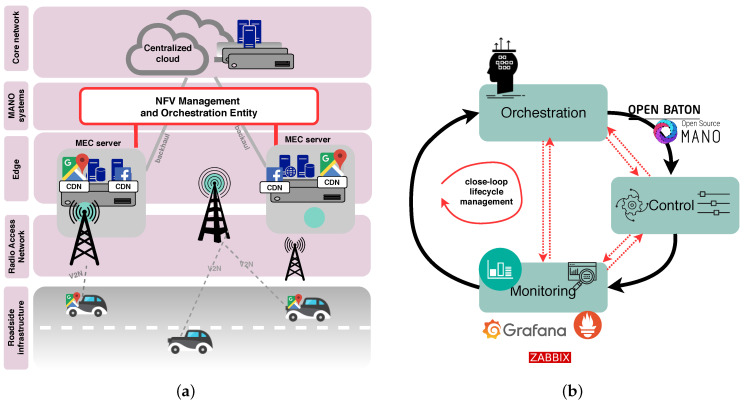
Management and orchestration in MEC-enhanced vehicular networks. (**a**) A high-level architecture of MEC-enhanced vehicular networks. (**b**) The closed-loop life-cycle management of network services.

Vehicular communications presently provide a support for a fruitful variety of safety (e.g., emergency electronic brake warning, lane change warning, forward collision warning, etc.), non-safety (e.g., traffic information systems), and infotainment (e.g., peer-to-peer gaming, IPTV, Internet content sharing, video streaming, etc.) vehicular applications [1,2,3,4,5]. Since the expectations towards vehicular communications are increasing, and ultra-low latency is a primary and critical concern for autonomous driving as an ultimate goal, there is an urgent need to leverage on emerging technologies such as 5G and Multi-Access Edge Computing (MEC) to facilitate the performance of various vehicular applications (Figure 1a). By jointly considering strict requirements for 5G networks, such as greater coverage, end-to-end latency less than 1ms, massive connectivity, and massive capacity, accompanied by enormous heterogeneity in network resources, technologies, vendors, operators, vehicles, etc., traditional manual network management becomes impossible to scale and to maintain. It is an utmost challenge to achieve high Quality of Service (QoS) and Quality of Experience (QoE) without sophisticated management and orchestration [6], which are, at the same time, compliant to the standards. Hence, such heterogeneity presses an urgent need for transition from a poorly scalable network management to its automation. In short, bringing 5G and MEC to vehicular networks reduces data transmission time [7] for latency-sensitive use cases such as autonomous driving, but requires automated network management in order to cope with aforementioned heterogeneity.

Therefore, in this paper, we study the automated closed-loop life-cycle management and orchestration of network services and resources within MEC, based on the three inter-coupled activities, i.e., orchestration, control, and monitoring (as shown in Figure 1b). The recent advances in Software Defined Networking (SDN) and Network Function Virtualization (NFV) aim to facilitate network management automation to a great extent when incorporated in MEC architectures. In particular, SDN and NFV bring more flexibility, and programmability to wired and wireless communication networks, while enabling higher resource use, and lower costs [8]. Yet, the full potential of such synergy is still to be discovered [6].

Figure 1a illustrates a high-level architecture of MEC-enabled vehicular networks, thereby spanning the vehicles themselves (with On-board Units (OBUs) equipped with sensors), Radio Access Network (RAN), edge, and the NFV Management and Orchestration (MANO) entity. Providing storage and computational resources at the edge, MEC is intended to reduce latency for mobile users (i.e., vehicles) by using more efficiently the mobile backhaul as well as the core networks [9]. The MANO manages and orchestrates MEC servers and the services deployed and running on top of these servers, and finally, the core network. The position of MEC platforms enables using resources exposed at the network edge to host, and to deploy numerous vehicular applications that can be easily instantiated, and terminated in a dynamic way. Furthermore, due to the opportunities to deploy vehicular services in a lightweight manner, MEC can also enable a migration of services from one machine that hosts the service to another if it is efficiently managed and orchestrated by a suitable MANO platform. In this way, a service migration tackles the low-latency requirements, since a new placement of services will ensure that low-latency can be achieved and maintained by responding to the vehicle movements in a proactive way.

The bottom level of the overall network snapshot depicted in Figure 1a presents an in-vehicle infotainment use case [3,4,5] in which vehicles exploit Content Delivery Network (CDN) as a service, with cache CDN servers placed within MEC in order to decrease the overall latency in accessing popular websites (e.g., Google maps). Such decentralized cloud architecture is going to be a cornerstone for vehicular communications, providing low latency services tailored to various 5G automotive use cases (e.g., active driving safety assistance, road traffic monitoring, cooperative manoeuvring, in-vehicle infotainment, emergency situations, etc.). Simultaneously, this cloud architecture assists in the offload of heavy computational tasks from autonomous vehicles to the edge.

In Figure 1b, we illustrate orchestration, control, and monitoring, as three parallel and interconnected processes. In particular, orchestration is a joint process of: (i) automation, (ii) coordination, and (iii) managing deployment and operation of network services. The role of SDN is to provide connectivity, and to keep a centralized abstract view of the network topology [8,10]. In the other hand, NFV is in charge of managing the network functions. With both support of SDN, and NFV, orchestration allows network services to be automatically deployed and managed [11]. In terms of control, we introduce existing MANO tools and exploit their control features. Finally, monitoring provides valuable input about available resources and network status to the orchestration entity, which can make decisions upon network services in a proactive and timely manner. Based on the decision made by orchestration entity, control tweaks the network service configuration, and performs resource re-allocation.

The synchronization between these three interconnected processes such as orchestration, control, and monitoring, is essential to enable automation for the resource and service management in strongly heterogeneous environments. Therefore, we map such closed-loop life-cycle automation onto the existing NFV MANO systems, and present perspectives on their incorporation within MEC-based vehicular networks.

Derived from the isolation of the most important Key Performance Indicators (KPIs) for automated management and orchestration, there are two major groups, i.e., feature-based and operational KPIs, which can be used to benchmark existing MANO solutions for supporting delay-sensitive vehicular applications. Accordingly, we summarize our contribution as follows:

1. We map the architecture of MANO solutions to the closed-loop life-cycle management and orchestration, pointing at its clear articulation in the research and industry fields.
2. A feature-based analysis: Taking into account the feature-based KPIs, i.e., key features of MANO tools (e.g., required resources needed to run a particular MANO, number of life-cycle management operations that MANO can effectively perform, etc.), we present a comprehensive overview of several MANO tools, developed within different research projects that recently increased the interest in network service orchestration. Due to their compliance to ETSI standards, and lightweight deployment opportunities, we see Open Source MANO (OSM) and Open Baton as suitable solutions for MANO operations within the resource-constrained network edge. Therefore, we bring their extensive performance analysis conducted in a real testbed setup as our next contribution.
3. A performance analysis: One of the common operational KPIs that is used to measure performance of MANO solutions is an overall instantiation delay. In particular, it refers to the time needed for a MANO solution to successfully instantiate a fully operational network service. Therefore, based on this KPI we evaluate the performance of Open Baton and OSM in a testbed environment, and discuss their comparison while providing instructions on their deployment in vehicular networks based on 5G and MEC. Besides this comparison, we showcase how in particular Open Baton responds to different virtualization technologies (i.e., containers and VMs) that are used for service virtualization. This extensive performance analysis is obtained in the high-performance testbed, mimicking the realistic features of edge computing in vehicular networks.

The paper is organized as follows: in the next section we present the background and related work. A thorough description on the closed-loop life-cycle management of network services in MEC-based vehicular networks is presented in Section 3. This is followed by an in-depth analysis of the features of existing MANO tools in Section 4, along with the performance analysis of Open Baton and OSM in Section 5. Finally, we conclude the paper and discuss future works in Section 6.

## 2. Background and Related Work

In this section, we provide an overview of works that motivated the incorporation of 5G and MEC to vehicular communications, aiming to achieve ultra-low latency as an ultimate goal. In Section 2.1 we overview existing works on leveraging MEC in vehicular context, while in Section 2.2 we present similar approaches to solve the insufficient flexibility and scalability of management and orchestration systems in MEC-enhanced vehicular networks. There is several works tackling MEC-based vehicular networks that are recently published, and within this section we present the ones which we consider important for the research direction of our approach.

### 2.1. MEC in Vehicular Context

A brisk overview of works that study the incorporation of MEC in vehicular communications is presented in Table 1. An example of the architecture of MEC support for vehicular communication systems is presented in Figure 1a, and the explanation of components of such architecture, as well as their relations is provided by Wang et al. [12]. To depict the peculiarities of such architecture, authors provided an architecture overview within confines of the two particular use cases, i.e., HD Map and Intelligent Intersection. In their tutorial-based approach, Shah et al. [1] present the mapping of the 5G key features on vehicular communications, explaining how these features can be beneficial for Vehicle-to-Everything (V2X) networks. As stated by Shah et al. [1], some of the major limitations imposed by IEEE 802.11p technology, which is currently a basis for Vehicle-to-Vehicle (V2V) and V2N, are: insufficient support for diverse applications (safety, non-safety, and infotainment), inadequate provisions for minimizing communication load caused by periodic beacons, ineffective congestion control, insufficient reliability, etc. Therefore, Shah et al. [1] point at the potential in using 5G on the way to resolving the aforementioned constraints. To enable the most various set of use cases, vehicular networks have to consolidate all the different requirements for diverse services (e.g., video streaming, cooperative maneuvering, lane change warning, etc.), and to foster the synergy between NFV, SDN, and MEC to meet these requirements.

According to Shah et al. [1], 5G and MEC are expected to improve the current support for vehicular applications, as most of them cannot be fulfilled only by Roadside Unit (RSU) connectivity. Since MEC relies on NFV to virtualize services, it is currently seen as a key platform for hosting diverse services, which can be discovered, accessed, and used, by vehicles [1]. To test a practical implementation of service provisioning in vehicular networks supported by edge and cloud, Laaroussi et al. [13] created an empirical analysis, by comparing the edge-based service provisioning, and the one provided by centralized cloud. Their results show that edge-based service provisioning outperforms the implementation with a centralized cloud in terms of achieved throughput, for the cases of different widespread application layer protocols, such as HyperText Transfer Protocol (HTTP), Constrained Application Protocol (CoAP), and MQ Telemetry Transport (MQTT).

Furthermore, Ning et al. [2] agree that despite the benefits brought by MEC (e.g., highly efficient usage of mobile backhaul networks) there is still a vast room for improvement on ubiquitous connectivity, energy-efficient computation, and ultra-low latency [2]. As it is estimated that around 45% of the generated data in 2020 will be processed by edge servers instead of centralized clouds [2], Ning et al. address the problem of offloading traffic from resource-constrained vehicles to MEC platform. They consider heterogeneous requirements of vehicle mobility and the computation tasks, integrating MEC-enhanced vehicular networks with Non-Orthogonal Multiple Access (NOMA) technology, which uses more efficiently the wireless spectrum [2].

As computation offloading is one of the benefits of bringing MEC to vehicular communications, there are numerous works that propose offloading schemes that optimize offloading decisions and resource allocation. However, task offloading supported by MEC is out of scope of our paper, and therefore more information can be found in [14,15,16,17].

### 2.2. Management and Orchestration of Resources and Services within MEC

In Table 2, we group background works into different categories based on whether they study orchestration, control, or monitoring, in a theoretical, or in a practical way. Regarding the MEC platform and its emerging features, Taleb et al. [9] present an extensive survey, which analyzes MEC as a decentralized cloud architecture that transforms the legacy Base Stations (BSs), into an IT environment at the edge of RAN. However, the orchestration of services and resources on a deployed MEC is recognized as a highly challenging task, due to the high speed of vehicles, and the need to maintain service continuity [9].

Furthermore, Soua et al. [6] discuss MEC in vehicular communications, as well as support that SDN and NFV provide to MEC, in order to meet the requirements of responsiveness, reliability, and resiliency for automated services. Within the literature scope that they spanned, Soua et al. [6] point at possible solutions for mobility-aware computation offloading, but they also focus on the resource management and orchestration challenges, mostly imposed by heterogeneity of resources and services at network edge. Soua et al. [6] and Taleb et al. [9] also discuss that this high heterogeneity in services, resources, technologies, and cloud infrastructure induce severe challenges to meet QoS and QoE requirements, and to maintain service continuity. Also, this heterogeneous nature makes the resource allocation [18] and management even more complex. Therefore, there is a need for more sophisticated framework for service and resource management, unifying networking and cloud orchestration [6,9]. Importantly, Soua et al. [6] accentuate the need to exploit the synergy between NFV, SDN, and MEC, to create a programmable, flexible, and controllable architecture, particularly customized for Cooperative, Connected, and Automated Mobility (CCAM) use cases. Such architecture leverages on deployment of SDN controllers and Virtual Network Functions (VNFs), which should consider traffic characteristics, wireless diversity, and mobility patterns. As presented by Abdelaziz et al. [8], such management of the traffic handling is possible by enabling standard interfaces of the control layer, which further makes application layer more flexible.

Furthermore, MEC can benefit from SDN and NFV because of the opportunity to facilitate management and orchestration, putting it into an software-based framework. As shown by Liu et al. [7], the control component of management in SDN-based vehicular network assisted by MEC, runs on the commodity operation system. Thus, the deployment, update, and administration can be implemented by a software procedure. As an example for such management and orchestration platform, Soenen et al. [19] thoroughly present their modular and programmable management and orchestration framework that can be tailored to a service, or a particular VNF. According to Soenen et al. [19], the aforementioned customization can be achieved by constructing the so-called function-specific managers, and service-specific managers. These managers should be described and configured within VNF Descriptors (VNFDs), and Network Service Descriptors (NSDs), so they support MANO entities towards managing and orchestrating a specific service and its resources in a custom manner.

Regarding the closed-loop life-cycle management and orchestration, there are several works which study concepts of the three constituent components (i.e., orchestration, control [20], and monitoring [21,22,23]), but only separately. Apart from specific vehicular-based perspective, de Sousa et al. [11] distinguish and present some key concepts of network service orchestration, and also provide an in-depth taxonomy of different orchestration approaches and solutions, paving the way for the realization of diverse orchestration application scenarios. From a theoretical point of view, to realize network service orchestration a Multi-domain Orchestrator (MDO) needs to be employed, as it coordinates resources and services in multiple administrative domains, spanning various technologies [11].

Moreover, both approaches presented in [9,11] provide a valuable overview of the research projects relevant for network service orchestration, altogether with several orchestration options that emerged from industry and standardization. In particular, de Sousa et al. [11] review the existing solutions from a more specific perspective than the general one provided in [9]. In their architecture-oriented overview, de Sousa et al. [11] studied the solutions based on the orchestration architecture, whether it spans one or multiple domains, in a hierarchical, cascade, or distributed manner, providing resource, service, or life-cycle orchestration, and so on. Regarding monitoring, there is an effort to study incorporating monitoring into the ETSI NFV architecture towards 5G, provided by Celdran et al. [21]. Their focus is on monitoring the control and data planes separately.

An interesting and yet realistic demonstration of using MEC for 5G connected cars is presented by Zhdanenko et al. [24]. This demonstration setup comprised cars, data collectors, analytical entities, and MEC orchestrator, showcasing also the impact of MEC server selection on the latency. In particular, the role of the data collectors is to aggregate the data from vehicles, such as GPS position and estimated changes in position over time. Furthermore, the analytical entities coordinate activities of all vehicles in their network in order to avoid collisions by pre-empting the next positions of vehicular traffic. The MEC orchestrator selects one MEC server based on any of the following criteria: (i) static cloud (no migration), (ii) distance-based MEC, (iii) load-based MEC, and (iv) distance and load-based MEC citeZhdanenko2019. The total delay as a KPI would depend on the decision that MEC orchestrator takes. However, in their demo-based paper, Zhdanenko et al. present only a high-level architecture of the aforementioned system, without getting into details about particular orchestration solutions.

One of the rare attempts to test MANO systems was introduced by Peuster et al. [25] recently. The main focus of their work is the test platform prototype that they developed to emulate up to 1024 Points of Presence (PoPs) on a single physical machine, which needs to be managed and orchestrated. Within the confines of their testing prototype, Peuster et al. [25] presented the concept of emulation-based smoke testing, used for automated, large-scale testing of two versions of OSM, i.e., OSM Release Three and Release Four.

Our approach to evaluate different MANO tools extends the perspective presented in the works we studied, since we: (i) map the architecture of MANO solutions to the proposed closed-loop life-cycle management and orchestration, emphasizing its importance towards automation of network service and resource management and orchestration in vehicular communications, (ii) provide an extensive analysis of the most used orchestration tools based on their features, and finally (iii) compare two widely recognized MANO solutions, i.e., Open Baton (Release Six) and OSM (Release Six), based on their performance in terms of instantiation delay, and their isolated features. The thorough feature and performance analysis that we performed tackles the suitability of these existing MANO solutions for orchestrating realistic latency-sensitive vehicular applications, and their readiness to respond to dynamics in vehicular environment.

## 3. The Closed-Loop Life-Cycle Management of Network Services in MEC

Within the confines of this section, we present our first contribution stated in the Introduction, i.e., we present our vision of automated closed-loop life-cycle management of network services and resources, and map ETSI NFV MEC MANO framework to this closed-loop. The demand for transition, from a traditional manual network service management toward automation, is described in terms of: (i) need to cope with strong heterogeneity in network resources, technologies, vendors, and operators, (ii) achieving ultra-low latency to fulfill strict requirements for various automotive use cases (such as active driving safety assistance, road traffic monitoring, cooperative manoeuvring, in-vehicle infotainment, emergency situations, among others), as high levels of other QoS and QoE parameters for vehicular applications, and (iii) being less prone to dynamic changes in mobile data traffic and radio conditions caused by high speed mobile users (i.e., vehicles). The consolidation of all the aforementioned strict requirements is a challenging task, pressing an urgent need for automation of network service and resource management and orchestration. As illustrated in Figure 1b, we present this phenomena in the form of closed-loop life-cycle management of network services as an essential synergy between: (i) Orchestration, (ii) Control, and (iii) Monitoring.

Although separated, these three branches are exceedingly dependent on each other, with the ultimate goal to facilitate the whole network service management process.

In particular, to make reliable decisions upon vehicular and network services, it is inevitable for the orchestration entity to receive a real monitoring report from the monitoring entity. Furthermore, control entities that implement orchestration decisions have to consider monitoring input in order to track the changes, and to tweak the network services’ configuration based on these changes. Therefore, to be able to extract the potential of each process, and to enable automation of MANO operations in MEC and 5G, the synchronization of these parallel, but interconnected, processes is inevitable. In this section we present each of these processes forming the closed-loop, and discuss the importance of their particular share in the automation of network service life-cycle management.

### 3.1. Orchestration and Control

**Figure 2:**
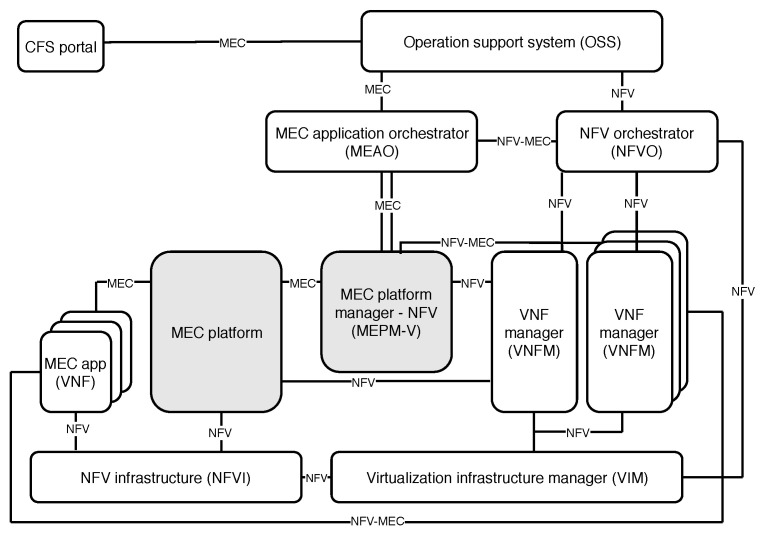
ETSI NFV MEC architectural framework.

**Figure 3:**
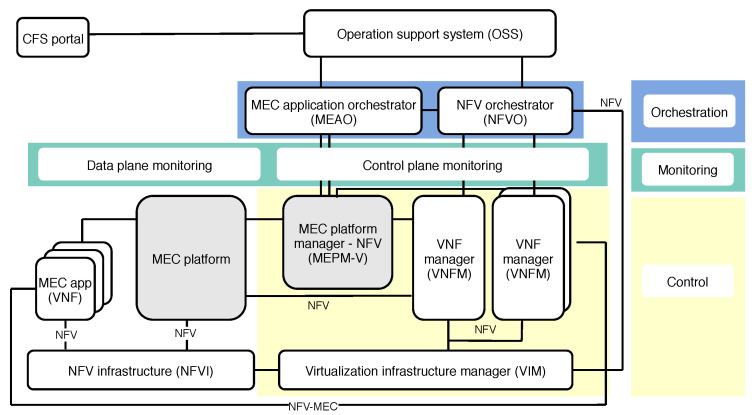
The closed-loop life-cycle managment of network services mapped to ETSI NFV MEC architectural framework.

To facilitate MEC’s incorporation into upcoming new generations of communication networks, and to enable better understanding of service and resource orchestration, ETSI formed an Industry Specification Group (ISG) to create a standardized and open environment, which enables the efficient and seamless integration of diverse applications from different vendors, service providers, and third parties [26]. ETSI NFV ISG defines NFV architectural framework, which is presented in Figure 2, altogether with the constituent elements and reference points needed for hosting applications within MEC platform. From the automated closed-loop life-cycle management perspective, such architectural framework is depicted in Figure 3, illustrating how we grouped the essential architectural elements into the orchestration, control, and monitoring categories. Therefore, due to their coexistence within ETSI framework, as well as their strong interdependence, the impact of orchestration and control is jointly discussed in this section.

The two main components of ETSI NFV MEC architecture are NFV Orchestrator (NFVO) and VNF Manager (VNFM), mutually assembling a so-called ETSI NFV MANO [27]. The NFVO, therefore, entails the orchestration functions, while VNFM stands for the control entity in charge of the life-cycle management of VNFs (i.e., VNF instantiation, scaling, terminating, etc.), as building blocks of the network services.

The advantage of this open source architecture lays in facilitated implementation of an NFV architecture, increasing the likelihood of interoperability among diverse NFV implementations. The last is particularly important to emphasize, since different MEC platforms comprise several virtualized and physical resources, diverse services, and applications of various stakeholders. In such strongly heterogeneous environment, interoperability plays a crucial role, which can be assured only by following the standardization guidelines and recommendations.

#### 3.1.1. Orchestration

The orchestration comprises processes of automation, coordination, and management of deployment and operation of network services [11]. In particular, the NFV architecture and orchestration framework proposed by ETSI establishes the following three domains: (i) VNFs, as software defined network functions, (ii) NFV Infrastructure (NFVI), consisted of hardware and software components for deploying VNFs, and (iii) NFV MANO providing organization and management of NFVI, which is responsible for the life-cycle management of VNFs, i.e., network services [9,20]. As Figure 3 clearly depicts, the orchestration in such architectural framework spans two different blocks, i.e., MEC application orchestrator and NFVO, which orchestrate life-cycle management operations of MEC applications and network services, respectively.

According to Taleb et al. [9], the true impact of MEC paradigm relies on the service orchestration capabilities as well as on the interaction with network architecture. Being aligned with ETSI NFV framework, MEC framework (Figure 2) includes virtualized infrastructure, as well as applications, and VNFs deployed on top of it. Taleb et al. [9] consider the service-related attributes such as: resource allocation, service placement, edge selection, and reliability, as of particular relevance for the efficient orchestration. In the context of resource allocation, Taleb et al. [9] provide an overview of research efforts to study how the efficient resource allocation strategies impact the overall process of orchestration. A strongly heterogeneous pool of resources (virtualized and physical) is present within MEC platforms, being allocated to serve various services and applications installed on top of the platforms. Hence, it is expected that the brain of the orchestration process—i.e., orchestrator, takes care of efficient resource use in order to meet stringent service requirements, such as those in vehicular networks. In the context of vehicular applications, MEC service placement and MEC server selection over different platforms is an utmost challenging task due to high speed mobility and use-case-dependent service deployments. It means that different stakeholders might be included in the service design and deployment, which depend on the specifications required by different use-cases. For instance, in cooperative maneuvering use-cases, multiple vehicles are being served by one or multiple MEC servers. The last case requires multiple instances of service being instantiated on each edge server, suitable for hosting application. Therefore, the orchestration is in charge of managing all service instances among different MEC platforms, in a manner which enables achieving corresponding QoS, QoE, and resource use.

In their survey on network service orchestration, de Sousa et al. [11] claim that the foundations of orchestration are routed back to the three enabling technologies, i.e., SDN, NFV, and cloud computing. In regards to that, they explain the interrelation between them stating that the SDN is in charge of enabling connectivity, NFV manages the network functions, while network service orchestration governs all the deployment processes of the end-to-end network service. According to the study presented by de Sousa et al. [11], orchestrators can be classified based on their functional scope, as follows: (i) service orchestrator—carries out service composition/decomposition, (ii) life-cycle orchestrator—manages the workflows, processes, and dependencies across service components, and (iii) resource orchestrator—maps service requests to resources, either virtual or physical. Another classification is provided based on the operational scope of the orchestrator. Accordingly, domain orchestrators have an absolute control over all resources that belong to their unique domains, but being limited to the administrative boundaries. On the other hand, multi-domain orchestrators have a broader scope but are therefore more complex, enabling end-to-end service orchestration while spanning different administrative domains [11].

#### 3.1.2. Control

The essential control blocks included in ETSI NFV architectural MEC framework are illustrated and emphasized in Figure 3. As already stated in the previous section, VNFMs are responsible for the VNF life-cycle management tasks including, for instance, its instantiation, scaling, pausing, restarting, and termination. However, VNFM is also in charge of reporting the VNF states to NFVO, so it can promptly react to changes, and make decisions on VNF placement and relocation. More so than ever, the dynamic changes in network traffic and service request patterns require continuous management of services, in terms of allocating more resources, VNF scaling up or down, releasing unnecessary resources, and terminating, with an ultimate goal to achieve or maintain satisfactory level of QoS, QoE, and resource use.

Besides VNFMs in the control entity shown in Figure 3, there is a MEC platform manager which: (i) manages installed MEC applications (e.g., vehicular applications), including informing orchestrator of relevant events from applications, (ii) provides element management functions to the MEC platform, and (iii) manages application rules and requirements (such as service authorization, traffic rules, etc.) [28]. Another role assigned to the platform manager is to control fault reports and performance measurements about virtualized resources, which are all collected by Virtualized Infrastructure Manager (VIM) and forwarded to the platform manager for further processing [28].

To enable development and deployment of VNFs, and MEC applications, controlled by VNFM and MEC platform manager, virtualized infrastructure consisted of computing, storage, and networking resources requires proper control as well. Therefore, VIM performs the allocation, management, and releasing of these resources, and prepares the underlying NFVI to run software images as basis for the required VNFs. As already mentioned, VIM also collects and reports performance and fault information about resources, delegating the reports to VNFMs. Importantly, once when it is supported by MANO systems, service relocation/migration will be performed by VIM [28].

### 3.2. Monitoring

As we identified monitoring as one of the three crucial segments of closed-loop life-cycle management of vehicular services in 5G networks enhanced by MEC, this section summarizes the main research efforts towards monitoring network services to improve management and orchestration efficiency. In general, the overall monitoring process has to ensure that each network service is running properly, by extracting the critical information from the physical or virtual nodes (i.e., network functions, links, etc.), and sending important notifications to the orchestration and control entities. It comprises data collection and information extraction, which are directly performed by monitoring entity shown in Figure 3. The extracted information is further leveraged by orchestration plane which makes corrective decisions. Afterwards, the control entity performs the actions implied from the orchestration decisions, which might include resource re-arrangement, VNF/service migration, scaling, and terminating.

The project 5GTango [22] has recently recognized the importance of having an adequate monitoring tool to be embedded into automated management system in 5G networks. Therefore, the project consortium [23] has identified several constraints of currently available monitoring tools, which limit their usage in 5G networks, as follows:

- intrusiveness for short-lived network function instances
- not being able to follow the pace of dynamic management
- not covering the requirements for both container-based and hypervisor/VM-based network function deployments
- not being suitable for collecting data from different cloud environments.

Taking into account the aforementioned characteristics which constrain monitoring of network services, incorporation of a monitoring tool with general purposes into the closed-loop life-cycle management of MEC-based vehicular services is not a straightforward task. Although theoretical, an effort to approach this problem is presented by Celdran et al. [21]. In their study of automatic monitoring for 5G networks, Celdran et al. [21] note that monitoring has to be included within automated management of 5G services, since otherwise managing monitoring of network services would be impossible to perform due to the enormous number of connected devices and their high mobility. The authors provide an important aspect for isolating the information which needs to be monitored, in order to provide necessary input for network service life-cycle management and orchestration with an ultimate goal to improve QoS and resource use.

Thus, there are two distinct types of information to be monitored: (i) Data-related Information (DRI), such as information contained in network flows, and (ii) Control-related Information (CRI) —i.e., users’ mobility, network infrastructure location, number of active users, percentage of CPU and storage consumed by the network service, etc. The CRI is of particular importance for ensuring the correct provision of monitoring network services, as it directly affects the network service orchestration process and the corrective actions that need to be derived. Therefore, Celdran et al. [21] propose a solution which incorporates monitoring into the architecture oriented toward 5G networks, which integrates SDN and ETSI NFV architectural proposal. To adequately manage the monitoring process, they propose to monitor control and data plane separately (Figure 3). With a specific focus on the control plane, i.e., gathering CRI, the architectural components (e.g., VNFM, VIM, SDN applications, etc.) expose the information to CRI monitoring component, which therefore aggregates all the upcoming information and forwards it to the decision making entities. In such asset, the monitoring on the VNF level can be performed, tweaking resources allocated to each VNF based on the decisions made in orchestrator.

Currently, there are various monitoring tools available for different purposes, and for instance, cloud monitoring has a resourceful research background. However, all of these tools are customized to the specific types of VIM (e.g., OpenStack [29], Amazon Web Services (AWS) [30], VMWare [31], OpenVIM [32], etc.), making them dependent on the specific virtualized infrastructure, which is hard to scale especially in such heterogeneous environment as MEC in 5G. For instance, the most popular monitoring solutions for OpenStack are Ceilometer and Nagios, which meter the data related to OpenStack resources such as compute, networking, and storage. In case of AWS, there is CloudWatch which monitors Amazon EC2 instances, Amazon RDS databases, and Amazon DynamoDB, and sets alarms with specific priorities based on the severity and importance of the information that is being monitored. Hamid and Shah [33] assess the performance analysis of the aforementioned types of monitoring tools, including vROPS which is used for monitoring VMWare resources. Their effort to integrate AWS monitoring support into the Open Source MANO orchestration tool is presented in [33], in which they elaborate on the idea to create an integral monitoring component which will consist of various plugins customized to different VIMs. In particular, they detail on how to create plugin for monitoring AWS resources, aiming to automatize the overall monitoring process by excluding the need for manual configurations. Such active monitoring of individual resources that belong to AWS cloud enables proactive and automated troubleshooting and self-healing of resources [33]. However, due to their strong dependence on the specific VIM types, the capabilities of available monitoring tools are limited, and therefore research in this field should be further intensified.

## 4. A Feature Based Analysis of Existing MANO Tools

As stated by our second contribution in the Introduction, in this section we present an extensive feature-based analysis of existing open source MANO tools, which are widely recognized in both academia and industry circles. Through a thorough examination and study of the available documentation and research papers that tackle a particular MANO tool, we isolated key features that need to be taken into account when studying these tools. We find such analysis as notably important for the future research in the field of resource and service orchestration, because it provides a summarized information on the tools which are likely to be used in the real deployment, and can be used as guidelines for future extensions of existing orchestrators. Each particular feature is essential to consider, as it highly affects the performance of the tools and their ability to get customized to different experimental environments.

Based on the work provided by Taleb et al. and de Sousa et al. [9,11], the open source tools that attracted significant attention in the past few years are Open Network Automation Platform (ONAP), Open Baton, Sonata (5GTango), OSM, Tacker, Cloudify, X-MANO, TeNoR, and Escape. Since the background information for each of these tools, such as the research projects in whose scope the tool was developed, is already presented in aforementioned work, here we do not present the specific project and tool details. Therefore, in Table 3, Table 4 and Table 5, we map the feature types to their corresponding metrics for each MANO tool that we took into consideration, and the brief discussion based on each feature is presented as follows.

Resource footprint: It embodies one of the fundamental requirements prior to experimenting with a MANO tool, because it presents the amount of resources (such as number of virtual or physical machines, RAM, number of vCPUs, storage, etc.) needed for the installation and proper work. To make the result comprehensible, we present three categories, i.e., light, medium, and heavy, and map the required resources to them as presented in Table 6. Concerning the resource footprint, the three categories presented within Table 6 can help readers to resolve where is a certain MANO solution positioned on the scale from being lightweight to resource-hungry. The categories are based on the number of virtual CPUs that each MANO solution requires for its proper work, as well as the optimal values of RAM and storage. For example, the light MANO solutions can be successfully deployed inside a VM on the host, while medium, and especially heavy solutions, in most cases require dedicated resourceful bare-metal servers to efficiently perform their tasks.

In Table 3 and its extension (Table 4), the resource footprint is shown for each tool. It can be seen that ONAP is the heaviest in terms of all three resource components, which is expected due to its extensiveness, strong credibility, and relevance for the industry as well. On the other hand, Open Baton and OSM offer two installation possibilities, i.e., minimal and full, which differ in number of supported components (e.g., NFVO, VNFM, drivers for monitoring plugins, drivers for different VIMs, etc.). However, it should be noted that MANO tools that connect to VIMs such as OpenStack, require additional machines to install VIM, which in particular needs 4 vCPUs, 8 GB RAM, and more than 80 GB of disk space per se.

Messaging bus: This specific component is essential for enabling either synchronous or asynchronous communication between different MANO components, offering message exchange in a reliable way. The overview of two widely used messaging buses that are also used within MANO solutions, i.e., RabbitMQ and ZeroMQ, is presented in Table 7. Table 7 depicts the main differences between these two messaging buses in terms of the message exchange mode, message protocol, the mode of queueing, and their complexity. In particular, a complexity refers to the source code of the messaging bus, i.e., the number of lines of code needed to realize routing, load balancing, and persistent message queueing. As it can be seen from the Table 3 and Table 4, the great majority of tools use RabbitMQ messaging bus, due to its powerful and flexible operation. RabbitMQ is an open-source general purpose message broker that implements a variety of messaging protocols, with Advanced Message Queueing Protocol (AMQP) among them. In MEC-based MANO case, RabbitMQ provides MEC applications with a platform to send and receive messages, connect to each other, and scale. It is performed through different versions of point to point, request/reply, and pub-sub communication style patterns, which enable publishers to send messages to exchanges (central nodes), and consumers to retrieve messages from queues [34]. Due to this simplistic operation mode which enables routing, load balancing, and persistent message queuing in terms of several lines of code, RabbitMQ is easy to use and deploy, and therefore, it is reasonable that most of the MANO solution developers opt for this messaging broker. However, it inevitably generates additional latency because of message queuing on a central node. In regards to that, ZeroMQ [35], engaged by Escape, is a lightweight substitute for RabbitMQ, as it especially addresses latency constrained networking scenarios such as autonomous driving. However, the increased complexity of this solution is a significant disadvantage in comparison with RabbitMQ. Taking into account the importance of low-latency for vehicular applications, decision upon messaging system should be taken with a prominent attention, studying and benchmarking both RabbitMQ and ZeroMQ to find a trade-off.

Infrastructure adaptation: Using the term infrastructure adaptation, we consider the capability of the MANO tool to adapt to different types of VIM. The more VIM drivers supported by tool, the more flexibility in experimentation and deployment is provided. This is significantly important since different VIM types are more or less complex than the other, and if diverse set of VIM drivers can be easily installed within MANO, it expands the possibilities to combine resources from different virtualized infrastructures. All studied tools support OpenStack, as a widely used software platform which offers a plethora of virtualized servers and other resources to customers. However, due to the increased complexity in configuring OpenStack to work with a particular MANO tool, the support for additional VIM drivers that can be easily configured (e.g., AWS) should be more accentuated and motivated.

Virtualization environment: Despite the enormous popularity of Virtual Machines (VMs), the container-based virtualization is now gaining momentum, due to its capability to share the host kernel with user-space isolation. There is already a solid research conducted on capabilities of both VM and container-based virtualization, studying the benefits and limitations of both [9,11]. Tackling the resource availability within the MEC platforms, which is limited compared to the large and resourceful data-centers, the lightweight virtualization, and orchestration solutions for small-size programmable devices are required. Delivering a lightweight deployment of services and applications, containerization seems to be the best candidate for deployment of emerging 5G technologies such as NFV and MEC [20]. Therefore, the MANO tools with support for a container-based virtualization are considered to be profoundly interesting for future MEC-oriented research. The aforementioned enables orchestration and management of the latency constrained applications, placed and deployed within the edge of the vehicular networks.

VNF life-cycle operations: Depending on the type of the MANO tool, a certain number of life-cycle management operations is supported. Keeping in mind ONAP’s superiority and extensiveness compared to other tools, a support for a plentiful set of operations is expected. If we tend to approach the study of tools with lower complexity and lighter installation, most of the remaining tools provide support for number of operations of similar scale. Importantly, all of the tools enable three fundamental actions, i.e., instantiation, scaling, and termination. In particular, instantiation and on-boarding operations are usually tightly coupled. On-boarding means transferring appropriate image file altogether with VNF Descriptor (VNFD) and Network Service Descriptor (NSD), from NFVO to VIM via VNFM. In that phase, VIM allocates resources required for such VNF and network service, based on the specified flavor. On the other hand, instantiation is represented as a phase of booting-up a system based on the received image, and installing all dependencies stated in descriptors, which are needed for VNF or network service to run properly. In case of scaling, more resources are needed than it was initially allocated by VIM. Thus, based on the instruction from NFVO, VIM re-allocates resources, and in case of termination it releases the resources.

VNF package: A VNF package includes a corresponding VNFD that will be used to describe a VNF, as a part of the service chain that orchestrator aims to launch on top of the virtualized infrastructure. Besides VNFD, which provides a broader communication compatibility among operators, there is an NSD as well, containing description of the whole network service. Depending on the tool, these descriptors are usually written following some of the well-known standards, such as: Topology and Orchestration Specification for Cloud Applications (TOSCA), Yet Another Next Generation (YANG), and Heat Orchestration Template (HOT). For instance, TOSCA is a standard used to specify services and their relations on a cloud computing view, while YANG represents a data modeling language for configuration and state data manipulated by the network configuration, designed by IETF. As with TOSCA, HOT in particular, describes the resources and the relationship among them. However, being much more generic and able to automate any application production process, TOSCA is widely used for describing VNFs and network services. Nevertheless, given the broad support and availability of all three standards, we consider TOSCA, YANG, and HOT suitable for the orchestration solutions that we tackle in this paper. Finally, besides descriptors, a VNF package usually includes a VNF image which needs to be available on the corresponding VIM, so that Element Management System (EMS) entities are provided with an adequate image type for launching VNF-customized VMs.

VNF healthy environment support: This feature is quite specific since it is only available in ONAP and Sonata, representing incorporation of VNF self-healing capabilities such as those provided by integrated validation tools. In case of large-scale usage of the tools in industry and production, such capability is essential.

Integrated monitoring system: Recalling the closed-loop life-cycle management, which was presented within Section 3, and mapped to the ETSI NFV MEC architectural framework, there is a huge potential in integrating a monitoring system into the MANO solution. Such possibility decreases the delay in communication between monitoring and orchestration and control entities, therefore providing real-time information gathered from the measurements. Although some of the tools (e.g., ONAP, Sonata, and Cloudify) incorporate a tool-customized monitoring systems into their architectures, most of the studied MANO solutions still require installing plugins for external monitoring (such as Grafana, Zabbix, etc.).

Feature palette: It comprises different capabilities that MANO tool can provide to the users once it is properly installed. The palette is usually reached through some tool-specific Graphical User Interface (GUI), and in most cases it shows the actions that can be taken during the VNF life-cycle management.

Interfaces: Almost all of the encompassed MANO tools provide work on the resource and service orchestration, specific component configuration, actions from the life-cycle management set, and various activities from the feature palette, through both GUI—usually represented as a dashboard, and a Command Line Interface (CLI). Understanding of all the processes of VIM registration, creating VNFDs and NSDs, on-boarding VNFs, launching network services, etc., is facilitated by providing a corresponding GUI, as it is more representative than a usual CLI. Although the installation of each tool must be obtained through the CLI, representing the feature palette within a GUI-based dashboard is a plus.

Operating system: This feature only reflects the requirements based on the fundamental operating system, required for installation and proper work of the MANO tool.

ETSI NFV MANO compliance: In general, in order to expand the exploitability of any software tool, whether it is MANO or not, the standardization plays a key role as it assures that the tool meets certain requirements that guarantee the proper work in various conditions. Having ETSI NFV MANO framework (Figure 2) as a reference, it is unlikely that a tool with no proper compliance will be considered to be a candidate for the resource and service orchestration in MEC-enhanced vehicular networks, because ETSI has a leader role in standardizing NFV and MEC. The necessity for standardization in aforementioned context is reasonable, especially because of the heterogeneity in MEC platforms. Therefore, although developed and deployed by different vendors/operators/application designers and developers, various MEC platforms and applications can be consolidated and able to cooperate if the standardization requirements are met.

Multi-domain support: The multi-domain capabilities represent a strong contributing factor to filter the orchestration solutions, being characterized by capabilities to establish a connection with MEC from the other domain using technologies such as OpenVPN, and to enable communication among the resources in different administrative and technological domains.

Multi-tenancy support: Due to the ubiquitous popularity of network slicing paradigm, being able to allocate different slices of network resources to different QoS and QoE requirements is useful for the experimentation.

## 5. A Performance Analysis of Existing MANO Tools

Linked to the third contribution point (presented in the Introduction), this section shows a performance analysis of two open source MANO solutions, i.e., Open Baton and OSM, aiming at inspecting their suitability for orchestrating realistic latency-sensitive vehicular applications. First, we outline the experimentation setup by presenting: (i) the type of network service that we used for testing, (ii) the metrics that we defined in order to evaluate the performance of MANO solutions, (iii) installation steps and setting-up environment, and (iv) description of testbed that we used for assessing a performance evaluation. Second, we present the results that we gathered during the measurement of the KPIs presented in Section 5.1.2, and discuss the results and point at the clear articulation of incorporating these MANO tools into the framework of automated closed-loop life-cycle management of vehicular services.

### 5.1. Experimentation Setup

#### 5.1.1. Network Service

As a service that needs to be dynamically instantiated, we chose CDN as a Service (CDNaaS), for infotainment purposes within a vehicular context (e.g., loading Google maps with reduced latency), thereby investigating whether MANO tools are capable of enabling dynamic service creation and management. As Taleb et al. presented in [36], CDNaaS represents a service instance of virtual CDN, with aim to strategically instantiate and place CDN VNF instances over the cloud/edge nearby users. This way CDN VNFs can be dynamically instantiated based on users’ needs, content popularity, viewers’ geographical distribution, mobility patterns, etc. Therefore, in both cases of OSM and Open Baton, we instantiate CDN VNFs as cache servers for a specific website (such as Google Maps), so the users get the website content with an expectedly lower perceptible latency. The motivation to experiment with such type of service is its particular edge-suitability, which means that dynamic instantiation of necessary CDN services significantly affects users’ latency [36,37,38,39]. As measuring latency at the user equipment side is out of scope of our paper, we leverage the results provided in [36,37,38,39], which show the latency-related benefits of deploying CDN at the network edge. Thus, the scope of our performance analysis is to measure overall instantiation delay, as the time needed for MANO system to instantiate a network service on top of MEC.

For the purpose of testing, we created four types of network services, i.e., Service Function Chains (SFCs). Each SFC consist of one or more VNFs that are chained in order to deliver the full functionality of a final network service. As presented in Table 8, SFCs that we created are differentiated by number of VNFs that they contain, i.e., they contain one, two, three, and seven VNFs, respectively. To create a fair environment for benchmarking MANO tools, we used the same types of network services for both tools, therefore, customizing VNF and network service descriptors, so they can be interpreted by both NFVOs. In case of Experiment 2, instead of VNF descriptors, we built a necessary Docker image, and made it available at NFVI, so Open Baton could make use of it while instantiating service in the form of container.

#### 5.1.2. Metrics

**Figure 4:**
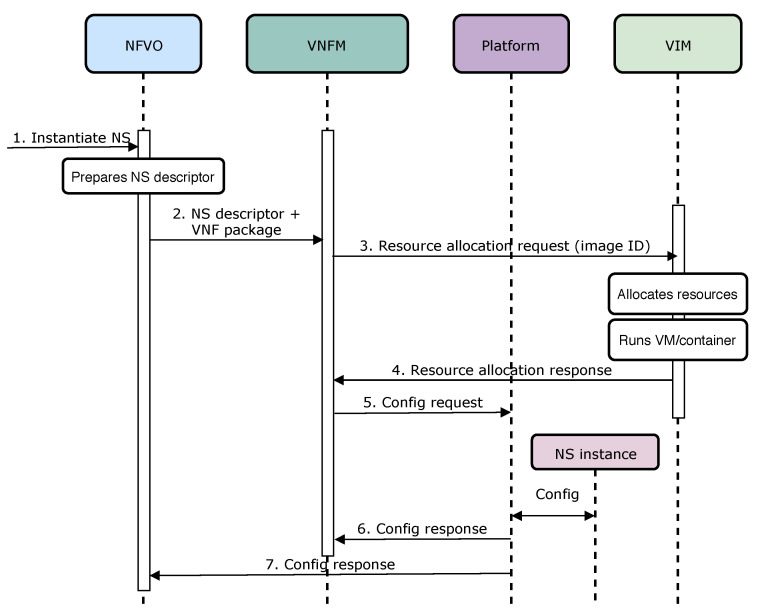
The process of instantiation of network service on top of the NFV infrastructure.

To assess performance evaluation of Open Baton and OSM, within the Experiment 1 we considered: (1) Overall Instantiation Delay (OID) of network service, and (2) CPU and RAM use. Accordingly, in the Experiment 2, we benchmarked container-based, and VM-based performance of network service instantiation, in terms of its duration, i.e., OID. As defined in Table 9, the instantiation delay is the overall time needed for a network service to be on-boarded and instantiated on top of the NFVI. To illustratively explain the aforementioned KPI, we created a sequence diagram ( Figure 4), which presents the communication between particular MANO components towards instantiating network service.

Therefore, OID is a particular metric that can be used to evaluate performance of MANO solutions, based on the time they need to on-board, and to instantiate a network service. Besides OID, we also measured CPU, and RAM use. In case of CPU, use is measured as an average usage of processing resources, i.e., the amount of work with which a MANO solution burdens the CPU of the underlying host. Accordingly, the usage of RAM means the average allocated memory needed for a MANO operation.

Some other metrics are run-time metrics that can be used to benchmark MANO’s performance during service execution, when it is up and running (e.g. scaling in and out). The run-time metrics are of high importance for MANO performance, as they directly contribute to perceivable KPIs by users. In particular, when more resources are needed for service operation, orchestrator should re-allocate resources, and scale-up ongoing network service in order to avoid potential service disruptions. However, although stated in their documentation that both MANO solutions support run-time operations, we revealed that it is not the case. Therefore, benchmarking of MANO solutions is limited on on-boarding and instantiation procedures for now.

#### 5.1.3. Installation and Environment Setup

To approach the experimentation, we created the two following experimental setups:

- Experiment 1: we provide a performance analysis of Open Baton and OSM, and compare them based on the overall VM instantiation delay, CPU, and RAM use,
- Experiment 2: we examine how Open Baton behaves when different virtualization technologies, i.e., Containers, and VMs, are used to instantiate network services.

To generate a fair environment for comparison of Open Baton and OSM, we created Experiment 1 in which we used OpenStack as a VIM for both MANO systems. As already shown in Table 3, OSM Release Six does not provide support for Containers as a virtualization technology, and therefore, Experiment 2 shows the performance analysis of Open Baton in case it instantiates network services as Containers, and VMs.

**Figure 5:**
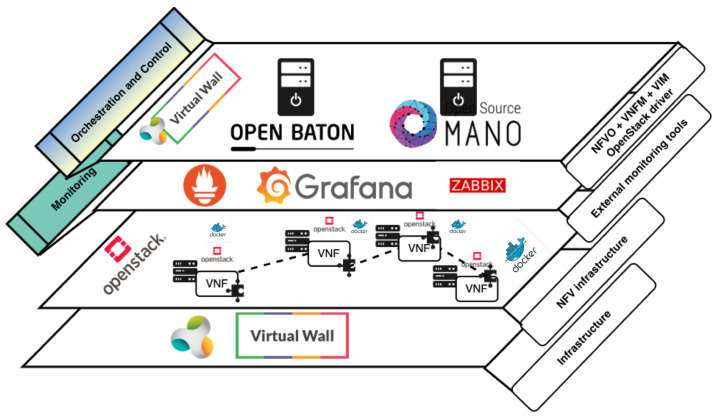
Experimentation setup on Virtual Wall testbed.

Regarding the overall experimentation setup, Figure 5 displays the MANO components which were deployed within both of our experiments, altogether with the software components that we used. In particular, the bottom layer is presented as NFV infrastructure, which hosts VNF chains, i.e., network services. As Figure 5 clearly depicts, we used OpenStack, and Docker, to make NFV infrastructure available for instantiating VNFs. Within the middle layer, Prometheus in collaboration with Grafana was used as an external monitoring tool for OSM, while Open Baton allowed monitoring via Zabbix external monitoring plugin. Finally, on the upper layer, Open Baton and OSM were installed and set up to embody the roles of orchestration and control. In Table 10, specific details on installation of Open Baton, OSM, and OpenStack, are provided.

Being aligned to Figure 3, and the way we mapped particular components of ETSI NFV MANO framework to closed-loop life-cycle management groups (i.e., orchestration, control, and monitoring), the upper layer in Figure 5 comprises both orchestration and control, which means that both processes are performed by MANO entities. Thus, Table 11 shows which MANO components belong to particular process.

The middle layer of experimentation setup in Figure 5 is in charge of monitoring tasks, which in collaboration with upper layer, closes the loop of automated life-cycle management of network services.

To realize orchestration of network services and resources, we considered tools with lighter installation setup, in order to create a lightweight orchestration environment, suitable for resource constrained MEC platform on the network edge. Due to the capabilities of similar scale (Table 4 and Table 5), we chose Open Baton and OSM for the experimentation and performance analysis. OpenStack is an open-source software platform for cloud computing, and MEC platform providers consider it as a suitable solution for enabling MEC infrastructure. Following this trend in both industry and academia, we installed OpenStack to provide underlying NFV infrastructure whose resources need to be orchestrated in order to properly host network services. On the other hand, Docker is a platform that enables developing and running the applications, while separating them from the infrastructure, so the software can be delivered quickly [40]. In our case, Docker used resources that were available within the NFV infrastructure on top of which it was installed and configured.

Both MANO solutions are open source platforms with a goal to provide a comprehensive implementation of the ETSI NFV MANO specification for orchestrating heterogeneous NFV infrastructures. Open Baton [41] is built by the Fraunhofer Fokus Institute and the Technical University of Berlin [11]. We installed the latest version which includes OpenStack VIM driver for deploying VNFs on OpenStack, generic VNFM for instantiation of VNFs, Fault Management System (FMS) for detection and recovery of VNF faults, Auto Scaling Engine (ASE) for automatic creation and termination of VNF instances, and Network Slicing Engine (NSE) for ensuring a specific QoS for a network slice (Table 10 and Table 11). OSM [42] is an ETSI-hosted project for delivering open source MANO tool, and the seventh release was recently launched. Its orchestration functions are divided into two entities: resource and service orchestrator. As presented in [11], OSM integrates several open source software initiatives to deliver fundamental ETSI NFV MANO functionalities. In particular, Riftware is used as a network service orchestrator, OpenMANO as resource orchestrator, and Juju Server as VNFM [11]. We installed OSM Release Six, which enabled the use of service and resource orchestrators, VNFM, OpenStack VIM driver, and fault management (Table 10 and Table 11).

**Figure 6:**
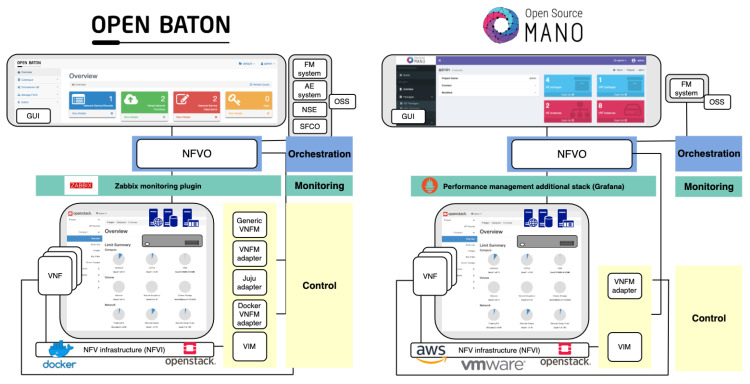
Open Baton and OSM architectures mapped to ETSI NFV MANO.

In Table 11, we map installed components of both MANO tools to the closed-loop life-cycle management and orchestration. A more illustrative representation of mapping Open Baton and OSM to closed-loop life-cycle management and orchestration, showing their compliance to ETSI NFV MANO framework at the same time, is presented in Figure 6.

#### 5.1.4. The Virtual Wall Testbed

For the experimentation setup, we used the Virtual Wall testbed, which is a large scale generic environment for advanced networking, distributed software, cloud, big data, and scalability research and testing [43]. In overall, the testbed contains more than 400 bare metal and GPU servers which are fully configurable in terms of their software installation, as well as the interconnection between network interfaces. Regarding connectivity, each node has a public IPv6 address as well as public IPv4, and thus can be easily accessible from any machine inside or outside of testbed environment. As nodes can be used for wide variety of purposes (such as terminal, server, network node, and impairment node), we used three of them to install OpenStack, as virtualization infrastructure, altogether with Open Baton and OSM, as MANO entities [43] (Figure 5). The Virtual Wall testbed is a part of FED4FIRE+ [44] project, which is the largest federation of next generation internet testbeds in Europe. Additionally, the testbed is powered by the jFed [45] experimentation toolkit that allows experimenters to push their code to the nodes. It offers to experimenters the possibility of experiment scheduling and a GUI with a real-time information of the experiment execution. jFed platform is supported by Linux Containers (LXC) to submit the code. As shown in Figure 5, we enabled NFV infrastructure resources on top of the testbed infrastructure, in order to be able to instantiate network services.

### 5.2. Results

**Figure 7:**
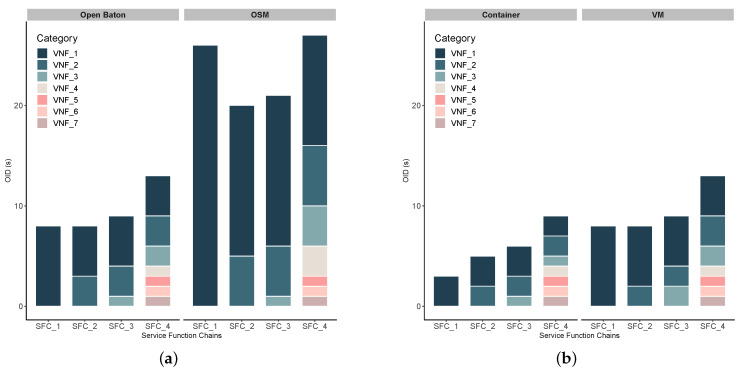
Management and orchestration in MEC-enhanced vehicular networks. (**a**) Network service instantiation delay: Open Baton vs. OSM. (**b**) Network service instantiation delay: Docker Containers vs. VMs.

**Figure 8:**
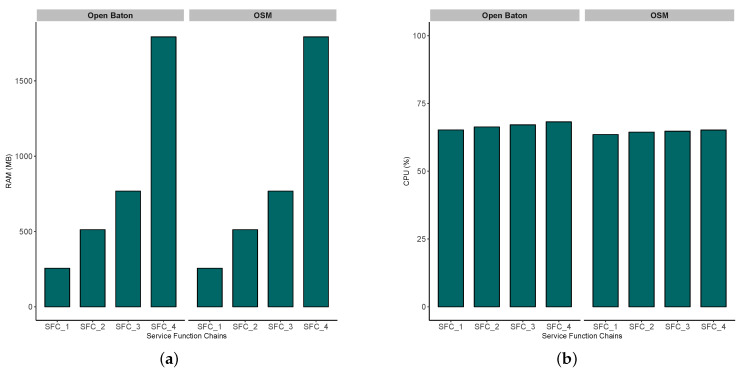
Management and orchestration in MEC-enhanced vehicular networks. (**a**) RAM use: Open Baton vs. OSM. (**b**) CPU use: Open Baton vs. OSM.

Regarding overall instantiation time, Figure 7a shows that performance of both tools highly depends on the number of VNFs chained into network service. In particular, Table 8 shows how are particular VNFs (from VNF_1 to VNF_7) connected to the service chains. If we examine the network service complexity, as several VNFs that a particular network service chain consists of, we notice the following:

- Open Baton outperforms OSM in case of service function chains with both lower and higher complexity (i.e., lower/higher number of VNFs in SFC). This statement is also supported by a statistical test, i.e., *t*-test, that is used for inspecting its statistical significance. Thus, we applied the *t*-test on the collected OID measurements for both Open Baton and OSM, and as a result we obtained $p_{value}=0.002192$. For the significance level of 95%, $p_{value}$ larger than 0.05 indicates acceptance of null hypothesis, i.e., the two samples are equal. Therefore, our result shows that the difference between measured OID for Open Baton and OSM is also statistically significant ($p_{value}<0.05$).
- In Figure 8a,b, the increasing trend from SFC_1 to SFC_4 is somewhat expected due the way how SFCs are generated (Table 8), i.e., the more VNFs are chained, the more memory and CPU resources are needed for an SFC to properly run. This trend has a lower slope in case of CPU, since CDN services that we instantiated as SFCs do not run CPU-intensive tasks. Furthermore, in CPU (Figure 8b) and RAM (Figure 8a) use results, we did not find significant difference between these two MANO tools, which was expected due to allocating the same flavors of VNF for both tools.

Within confines of the aforementioned observations, we can derive the following conclusions, as perspectives for incorporating Open Baton and OSM into real use-cases of automated closed-loop life-cycle management in MEC-based vehicular networks.

1. Taking into account a feature-based analysis presented in Section 4 and Table 3, OSM provides a lightweight solution for orchestration of network services and resources, as it requires much lower capabilities than Open Baton. Such advantage makes OSM more suitable for installation and setup on resource constrained edge cloud platform, such as MEC.
2. Regarding compatibility with different VIM environments, the OSM Release 6 supports more VIM drivers than the last version of Open Baton. Thus, the possibilities of customizing OSM to various NFV infrastructure types are broader than in Open Baton.
3. Based on our experience during the experimentation, both tools suffer from insufficient and inconsistent documentation, which complicates the overall process of installation and setting up.
4. As we already emphasized in Section 4, the support for container-based virtualization is important if we take into account the limited resource availability in MEC platforms. Open Baton supports containerized network services and applications, which is a significant advantage over OSM. Although the latest release of OSM supports Kubernetes [46] as VIM, and accordingly enables containerized service deployment, it is in an early stage, and requires more testing.
5. Aligned to the previous point, results from Experiment 2 shown in Figure 7b show that container-based service instantiation takes less time for each service type, as expected due to the lightweight capabilities of Containers in comparison to VMs. Furthermore, in order to inspect the statistical significance of our results, we applied the *t*-test on the collected measurements for OID. The test resulted in $p_{value}=0.004332<0.05$, which indicates that the difference between OID values for Docker containers and VMs (instantiated upon Open Baton’s guidance), is also statistically significant. The difference in overall delay between corresponding container and VM variants are even larger that presented in Figure 7b, because after on-boarding and instantiation procedures, container-based service is ready to be consumed by users, while VMs instantiated on top of OpenStack only got their resources and IP addresses, but the automated configuration of underlying operating system takes 2–3 min more.
6. In both Figure 7a,b, we present the values of OID for each SFC as a stacked value, i.e., we show how each of the VNFs (from VNF_1 to VNF_7) contributes to the overall OID, needed for this SFC to be instantiated. In particular, if we take a look at the time needed for SFC_4 to be instantiated, we can see that VNF_1 contributes to the overall OID the most, while the last three VNFs (i.e., VNF_5, VNF_6, and VNF_7) take the least time for their instantiation. It can be depicted in both Figure 7a, and Figure 7b that the impact of the first VNF in the chain on the overall OID is the highest. However, such result is reasonable, and expected, as each of the VNFs are spawned by using the same image, which means that the on-boarding procedure is included in the instantion of VNF_1, and once it is instantiated, all the remaining VNFs will take much less time, since the image is already available to the VIM.
7. From the perspective of overall instantiation delay, we expect that Open Baton will enable more suitable environment for realistic vehicular service implementations, consisted of multiple more or less complex VNFs. As we already elaborated on importance of Ultra-Reliable Low-Latency Communication (URLLC) in automotive use cases, more attention should be paid to prompt service instantiation. However, although lower in case of container-based deployment, instantiation delay for Open Baton is still perceptible, and some pre-emptive methods for predictive instantiation are needed, so the services can be ready on a MEC platform at the moment when they are needed.
8. Taking into consideration all findings based on a realistic example of CDNaaS, none of these two versions of MANO tools are ready to be used in realistic scenarios for vehicular communications, as run-time operations such as service scaling-in and out, muting, migration, etc., are not supported yet.

From the conclusions presented above, we see that selecting a MANO tool is not a straightforward task. Different tools provide sets of multiple benefits, depending on the perspective we have. Therefore, we presented perspectives for using these two particular MANO tools in MEC-enhanced vehicular communications. The feature-based and performance analysis that we provided in this paper, are valuable for both academia and industry, and provide guidelines on facilitated incorporation of closed-loop life-cycle management in vehicular networks based on 5G and MEC. Additionally, having extensive feature-based and performance analysis presented in this paper, our analysis can significantly facilitate development of new MANO tools. Since we focused on Open Baton and OSM for the performance analysis of MANO solutions, as these are the most suitable solutions for resource and service management and orchestration within the network edge, our future work will expand the performance evaluation setup, considering more MANO tools (e.g., a comprehensive tool such as Sonata—5GTango). In addition, we plan to measure the impact of different MANO tools on the delay measured at the user equipment, i.e., taking into account the dynamics in network as well. To this end, we aim at chaining the MEC services to virtualized RAN functions (e.g., OpenRAN paradigm), thereby performing the closed-loop life-cycle management operations on the configurable service chains from the edge to the user equipment, making these services tailored to the users’ needs. Furthermore, we are particularly interested in evaluating performance of different tools based on the service migration, and other run-time operations, once they are supported and well documented in the existing MANO solutions.

## 6. Conclusions

To cope with strong heterogeneity in resources, services, vendors, etc., as well as high dynamicity in network traffics, followed by high mobility of users in vehicular communication presently, automation of network service management and orchestration can come up as a solution. As a study to exploit the features of network management and orchestration aiming to support delay sensitive applications, in this paper we presented the closed-loop life-cycle management of network services as an essential collaboration between orchestration, control, and monitoring. Furthermore, we created a comprehensive feature-based analysis of the most adopted existing MANO solutions. Finally, we extensively evaluated the performance of Open Baton and OSM, recognizing the main components of closed-loop life-cycle management in their MANO architectures. Having latency as a crucial parameter for all latency sensitive vehicular applications, we assessed the overall delay in service instantiation, in order to explore the contributing factor to overall latency that needs to be minimized. Regarding the latency requirements at the user equipment side, we further study the benefits of bringing CDNs to the network edge by leveraging existing works and, in order to benchmark different MANO tools at the network edge, we measure the service instantiation delay of each solution. Based on the features and performance analysis of MANO tools, we presented valuable perspectives for incorporating MANO tools to realistic MEC-enhanced vehicular network scenarios. Taking into account both feature-based perspective and performance, our thorough analysis of OSM and Open Baton shows that Open Baton outperforms OSM in case of delay in instantiating CDNaaS instances. In case Open Baton deploys network service as a Docker container, it significantly reduces the duration of the overall instantiation process. Nevertheless, due to the limited support for service scaling, and migration, none of these two particular versions of MANO solutions has reached a level of maturity to adequately respond to dynamics in realistic vehicular applications.

Therefore, our future work will include more MANO solutions (e.g., a comprehensive tool such as Sonata—5GTango) in performance analysis, and extended number of run-time metrics, once they are fully supported. Furthermore, we plan to measure the delay at the user equipment, i.e., inspecting the dynamics present in network. This research aims at chaining the MEC services to virtualized RAN functions, thereby performing the closed-loop life-cycle management operations on the use-case tailored service chains from the edge to the realistic user equipment, such as real vehicles.

## Figures and Tables

**Table 1:** 5G and MEC in vehicular context.

Research Direction		Work
5G in vehicular communications		[1,12]
MEC in vehicular communications	MEC and Non-Orthogonal Multiple Access (NOMA)	[1,2,12,13]
	Computation offloading to MEC platforms	[1,12,13,14,15,16,17]

**Table 2:** Management and orchestration of resources and services within MEC.

Research Direction	Approach	Processes		Work
Closed-loop life-cycle management and orchestration	Theoretical	Orchestration	Challenges (vehicles’ high speed, service continuity, high heterogeneity)	[6,8,9,11]
			Programmable software framework for management and orchestration (SDN, NFV, MEC)	[6,7,8,11,18,19]
			Overview of research projects	[9,11]
		Control		[20]
		Monitoring		[21,22,23]
	Practical	Orchestration	MEC for 5G connected cars	[24]
		Control
	
		Monitoring	MANO evaluation	[25]

**Table 3:** A feature-based analysis of existing ETSI NFV MANO systems—part 1.

Feature Type		ONAP	Open Baton	Sonata	OSM
	number of vCPUs	heavy	minimal version: light full version: heavy	light	minimal version: light full version: light
Resource footprint	RAM	heavy	minimal version: light full version: heavy	medium	minimal version: light full version: medium
	storage	heavy	minimal version: light full version: light	heavy	minimal version: light full version: medium
Messaging bus		Microservice Bus	RabbitMQ	RabbitMQ	RabbitMQ
Infrastructure adaptation (VIM)		OpenStack, Azzure, VMWare, and Wind River	OpenStack, Amazon, Docker, Test	OpenStack, Kubernetes, Sonata Emulator	OpenStack, VMWare, AWS, OpenVIM
Virtualization environment		VMs (currently)	VMs and containers	containers	VMs
VNF life-cycle operations		1. instantiation	1. instantiation	1. placement	1. modelling
		2. configuration	2. configuration	2. on-boarding	2. on-boarding
		3. elastic scaling	3. starting	3. instantiation	3. NS creation
			4. stopping	4. scaling in/out	4. NS operation
		4. automatic recovery from resource failure	5. terminating	5. termination	5. NS finalization
			6. scaling-in		
VNF package	VNF descriptor	TOSCA, YANG	TAR, CSAR (TOSCA)	domain specific language similar to TOSCA and HOT	YAML-based documents
	VNF image	N/A	QCOW work in progress	N/A	QCOW
VNF healthy environment support		various packaging and validation tools available and integrated	no	yes	no
Integrated monitoring system		yes	no, connecting to various systems via plugin mechanism (Zabbix plugin)	yes (advanced real-time monitoring system)	no, plugins for different VIMs available
Feature palette		1. deployment	1. deployment	1. life-cycle management of NSs, slices, VNFs	1. NS/VNF on-boarding
		2. configuration,	2 managing PoPs	2. management of SLA	
		3. monitoring	3. catalogue	3. performing VIMs, WIMs, end Endpoints	
		4. restart	4. marketplace	4. monitoring KPIs	2. lifecycle
		5. clustering and scaling	5. launching NSD	5. catalogue	
		6. upgrade	6. on-boarding NSD	6. specifying QoS requirements links	3. fault and performance management
		7. deletion			
Interfaces		Portal, Dashboard, Use case UI, External APIs, CLI	Dashboard (GUI), CLI	Portal (GUI), WEB interface, CLI	Dashboard (GUI), WEB interface, CLI
Operating system		Ubuntu	Ubuntu 14.04/16.04	Ubuntu	Ubuntu 16.04

**Table 4:** A feature-based analysis of existing ETSI NFV MANO systems—part 2.

	MANO System	Tacker	Cloudify	X-MANO	TeNoR	Escape
Feature Type						
	number of vCPUs	medium	medium			
Resource footprint	RAM	medium	medium			
	storage	heavy	light-heavy	N/A	N/A	N/A
Messaging bus		RabbitMQ	RabbitMQ	RabbitMQ	RabbitMQ	ZeroMQ
Infrastructure adaptation		VIM: OpenStack and Kubernetes	VIM: AWS, Azure, OpenStack, Vsphere	N/A	VIM: OpenStack, Open Daylight	VIM: OpenStack
Virtualization environment		VMs and containers	VMs and containers	VMs	VMs	containers
			1. event-stream processing	1. creation	1. start	1. initiate/start/stop NF
			2. metrics queueing		2. stop	2. connect/disconnect
			3. aggregation	2. chaining	3. restart	
					4. scale-in	
VNF life-cycle operations		N/A	4. analysis, etc.	3. deletion	5. scale-out	3. NF to/from switch
VNF package	VNF descriptor	TOSCA	TOSCA	JSON file, multi-domain NS descriptor: YAML	HOT	YANG
	VNF image	N/A	QCOW	N/A	N/A	N/A
VNF healthy environment support		no	no	no	no	no
Integrated monitoring system		no, drivers for Aodh, and Ceilometer	yes	no, plugin for Zabbix	no, plugin for VIM monitoring and Apache Cassandra	no
			1. uploading and deleting blueprints			1. SDN domain manager
			2. keep a directory of blueprints		1. NS/VNF Monitoring	
		1. VNF Management: VNF Catalog and VNFM	3. create multiple deployments for each blueprint,	1. VNF catalogues		2. Internal domain manager
			4. execute workflows		2. NS/VNF provisioning	
			5. execute healing and scaling			3. Remote domain manager
			6. view application’s topology	2. NS management panel		
			7. retrieve events		3. Service Mapping	4. OpenStack domain manager
			8. use plugins			
			9. view metrics			
Feature palette		2. NFV Orchestration: VIM Management, VNFFG Catalogue, VNFFG Manager, NS Catalogue, NS Manager	10. search logs	3. statistics panel (visualize and export collected monitoring information)	4. SLA Enforcement	5. Universal Node Domain manager
Interfaces		Horizon and CLI	CLI, WEB UI	Customer portal (GUI)	N/A	REST-API, GUI
Operating system		1. CentOS, Redhat 2. Debian and Ubuntu	1. RHEL/CentOS 6.x 2. RHEL/CentOS 7.x 3. Ubuntu 14.x/16.x/18.x 4. Windows 2008 and later	Ubuntu 14.04 LTS, Windows 8.1 and Windows 10	Ubuntu 14.04	Ubuntu 16.04

**Table 5:** A feature-based analysis of existing ETSI NFV MANO systems.

	MANO System	ONAP	Open Baton	Sonata (5G Tango)	OSM	Tacker	Cloudify	X-MANO	TeNoR	Escape
Feature Type										
ETSI NFV MANO compliance	NFVO	yes	yes	yes	yes	yes	not fully	yes	yes	yes
	VNFM	yes	yes	yes	yes	yes	not fully	yes	yes	no
Multi-domain support		yes	no	no	no	no	no	yes	yes	yes
Multi-tenancy support (Network slicing)		yes	yes	yes	yes	no	no	N/A	N/A	N/A

**Table 6:** Resource footprint categories for MANO tools.

	*Light*	*Medium*	*Heavy*
**number of vCPUs (N)**	2≤ N ≤4	4< N ≤8	N >8
**RAM** (R)	R ≤4 GB	4 GB < R ≤8 GB	R >8 GB
**storage** (S)	S ≤20 GB	20 GB < S ≤40 GB	S >40 GB

**Table 7:** Overview of messaging buses.

Messaging Bus	Message Exchange	Message Protocol	Queueing	Complexity
**RabbitMQ**	synchronous/asynchronous	Advanced Message Queueing Protocol (AMQP)	via centralized node	low
**ZeroMQ**	asynchronous	ZeroMQ Message Transport Protocol (ZMTP)	decentralized	high

**Table 8:** Types of service function chains.

Network Service (Service Function Chain)	Number of VNFs in the Chain	VNFs
SFC_1	1	VNF_1
SFC_2	2	VNF_1, VNF_2
SFC_3	3	VNF_1, VNF_2, VNF_3
SFC_4	7	VNF_1, VNF_2, VNF_3, VNF_4, VNF_5, VNF_6, VNF_7

**Table 9:** Overview of metrics.

Metric	Definition
Overall instantiation delay (OID)	The overall time needed for a network service to be on-boarded and instantiated on top of the NFV Infrastructure (NFVI).
CPU use	The average usage of CPU processing resources, i.e., the amount of work with which a MANO solution burdens the CPU of the underlying host.
RAM use	The average allocated memory needed for a MANO operation.

**Table 10:** Overview of installation within experiment.

Component	Type of Mmachine in Virtual Wall	Capabilities			Operating System
		RAM	CPU	Storage	
OpenStack	pcgen4	48 GB	2 × 8 core Intel E5-2650v2 (2.6 GHz)	250 GB	Ubuntu 18.04
OSM	pcgen5	16 GB	1 × 4 core E3-1220v3 (3.1 GHz)	250 GB	Ubuntu 16.04
Open Baton	pcgen5	16 GB	1 × 4 core E3-1220v3 (3.1 GHz)	250 GB	Ubuntu 16.04

**Table 11:** The closed-loop life-cycle management of network services mapped to MANO solutions.

MANO	MANO Components		
	Orchestration	Control	Monitoring
		OpenStack VIM driver	
		Generic VNFM	
Open Baton	NFVO	Fault management system	Zabbix plugin
		Auto-scaling engine	
		Network slicing engine	
	Resource orchestrator	OpenStack VIM driver	
OSM	Service orchestrator	VNFM	Performance management
		Fault management	

## Abbreviations

The following abbreviations are used in this manuscript:

AMQP	Advanced Message Queueing Protocol
ASE	Auto Scaling Engine
AWS	Amazon Web Services
BS	Base Station
CCAM	Cooperative, Connected, and Automated Mobility
CDN	Content Delivery Network
CDNaaS	CDN as a Service
CLI	Command Line Interface
CoAP	Constrained Application Protocol
CRI	Control-related Information
DRI	Data-related Information
EMS	Element Management System
FMS	Fault Management System
GUI	Graphical User Interface
HTTP	HyperText Transfer Protocol
HOT	Heat Orchestration Template
ISG	Industry Specification Group
KPI	Key Performance Indicator
MANO	Management and Orchestration
MDO	Multi-Domain Orchestrator
MEC	Multi-Access Edge Computing
MQTT	MQ Telemetry Transport
NFV	Network Function Virtualization
NFVI	NFV Infrastructure
NFVO	NFV Orchestrator
NOMA	Non-orthogonal Multiple Access
NSD	Network Service Descriptor
NSE	Network Slicing Engine
OBU	On Board Unit
OID	Overall Instantiation Delay
ONAP	Open Network Automation Platform
OSM	Open Source MANO
QoE	Quality of Experience
QoS	Quality of Service
RAN	Radio Access Network
RSU	Roadside Unit
SDN	Software Defined Networking
SFC	Service Function Chain
TOSCA	Topology and Orchestration Specification for Cloud Applications
URLLC	Ultra-Reliable Low-Latency Communications
V2N	Vehicle to Network
V2V	Vehicle to Vehicle
V2X	Vehicle to Everything
VIM	Virtualized Infrastructure Manager
VM	Virtual Machine
VNF	Virtual Network Function
VNFD	VNF Descriptor
VNFM	VNF Manager
YANG	Yet Another Next Generation
ZMTP	ZeroMQ Message Transport Protocol
